# Impact of Raising Awareness and Providing Feedback on Compliance to Antibiotic Prescription Guidelines in Pediatric Inpatients

**DOI:** 10.7759/cureus.51766

**Published:** 2024-01-06

**Authors:** Siddhi Hembade, Madhuri Engade, Avinash L Sangle

**Affiliations:** 1 Pediatrics, MGM Medical College and Hospital, Aurangabad, IND

**Keywords:** antibiotic selection, compliance to guidelines, quality improvement research, antibiotic stewardship program, antibiotic resistance

## Abstract

Introduction: Antibiotics are vital in managing infectious diseases that significantly burden health infrastructure in a developing country like India. However, the widespread and irrational use of antibiotics has given rise to the menace of antibiotic resistance that threatens to take us back to the pre-antibiotic era. Our study aimed to evaluate the baseline compliance to antibiotic policy in the pediatric inpatient ward and analyze the impact of interventions on compliance with the policy.

Materials and methods: The prospective study was done at MGM Medical College and Hospital, Aurangabad. The study included infants and children from one month to 18 years of age admitted to the pediatric ward. Patients' prescription charts were evaluated in 375 patients during the first three months of the study, and prescribed antibiotics were recorded and compared with standard treatment guidelines. The intervention included awareness, educational, and feedback sessions regarding antibiotic prescription policies. The antibiotics prescribed were analyzed in 375 patients during the next three months.

Results: We found out that in the pre-intervention and post-intervention phases, out of a total of 375 patients, 60% and 46.1% were on antimicrobials, respectively. Out of those who were on antimicrobials, only 46% were compliant with the policy initially. That increased to 61% after the intervention.

Conclusion: Awareness, education, and feedback regarding antibiotic prescription policy as an intervention helped increase compliance, though not to the desired level of more than 90%. Continuous cycles of awareness and feedback help achieve better compliance.

## Introduction

Infectious diseases are a significant cause of morbidity and mortality in a developing country like India, especially in the pediatric population. Antibiotics have been a vital asset in the management of infectious diseases. Antibiotic overuse is responsible for the emergence of multidrug-resistant infectious microorganisms. The treatment options for infectious diseases are depleting fast as the new antimicrobial drug development is slow, whereas the menace of antibiotic resistance is rapidly progressing [1-3].

High prevalence of methicillin-resistant Staphylococcus aureus, multidrug-resistant Enterobacteriaceae and Klebsiella pneumoniae due to extended beta-lactamases production, ciprofloxacin-resistant Salmonella, ciprofloxacin-resistant Pseudomonas aeruginosa, and many other microorganisms with drug resistance have been reported from India [2-4]. The consumption of vast spectrum and newer antibiotics has been reported to be high in India, as reflected by Kotwani and Holloway's study from New Delhi, which reported very high consumption of antibiotics in private clinics, pharmacies, and public sector patient care centers [5].

Indian Council of Medical Research (ICMR), considering the alarming rise in antimicrobial resistance and hospital infections, initiated the antibiotic stewardship, prevention of infection, and control program [6]. In 2014, the Indian Academy of Pediatrics, in collaboration with the ICMR, designed a four-point plan for tackling the issue of antimicrobial resistance that included the development and dissemination of national antibiotic guidelines for children, educating doctors and the public on rational use of antibiotics, development of infection control guidelines for smaller hospitals and nursing homes, and ensuring compliance and collection and collation of data on antibiotic resistance [7]. With this perspective, the present study was done to identify the baseline compliance to antibiotic policy in the pediatric inpatient ward and evaluate the impact of responsive interventions on compliance with policy.

## Materials and methods

The prospective study was done at the pediatric ward in MGM Medical College and Hospital in Aurangabad, Maharashtra. The study was from December 1, 2019, to August 30, 2021. Infants and children from one month to 18 years of age admitted to the pediatric ward were included in the study. The children who were daycare patients and those who needed neonatal intensive care unit (NICU) or pediatric intensive care unit (PICU) were excluded from the study. The present prospective study was done to assess the compliance of antimicrobial policy and evaluate the impact of an awareness program regarding rational antibiotic prescription as per ICMR and Indian Academy of Pediatrics guidelines. All patients admitted for diagnosis and initiation of treatment in the pediatric ward were enrolled (excluding those who required or were transferred out from the PICU and NICU). Each patient’s drug chart was reviewed daily from Monday to Saturday; data on each anti-infective prescribed and the corresponding documented indication was collected over a nine-month study period and entered into a Microsoft Excel (Microsoft Corporation, Redmond, USA) database. Baseline data was collected from December 2019 to February 2020 before the introduction of interventions. 

Definition of compliance 

The antibiotic prescription was defined to be compliant with the policy if (i) the documented clinical condition and the prescribed antibiotic matched the empirical pediatric antibiotic policy or (ii) the documented clinical condition and prescribed antibiotic(s) matched documented recommendations from an infectious disease/microbiology specialist [8]. The program included three phases, i.e., pre-intervention, intervention, and post-intervention. In the pre-intervention phase, baseline data was collected from 375 patients for three months starting December 2019. The intervention phase was planned for March to May 2020 and the intervention phase from June to August 2020; however, due to the COVID-19 pandemic, the intervention and post-intervention phases were conducted from March to May 2021 and June to August 2021, respectively. 

The intervention phase was carried out in three overlapping phases: awareness, education, and feedback on results. The awareness phase included policy awareness. During the first phase (March 2021), a multi-pronged awareness campaign was carried out, which comprised of (i) the distribution of pocket-sized cards presenting a summary of the policy to the junior residents; (ii) the display of policy posters on ward notice boards and health records trolleys; (iii) A series of announcements were made at clinical team meetings about the new policy; and the addition of the policy to induction literature for medical staff. 

In the education phase (April 2021), an education program was carried out, which included teaching sessions for junior doctors. Education and training about the antibiotic interventional program were carried out. Four teaching sessions were conducted on Saturdays using audio-visual and interactive methods.

The feedback phase (May 2021) was introduced with weekly feedback on policy compliance rates. Weekly feedback comprised a single-page summary showing (i) the number of admitted patients on antimicrobials, (ii) the percentage of patients prescribed antimicrobials with an indication recorded in the health record, and (iii) the percentage of patients compliant with the anti-infective policy (iv) length of stay. This summary was sent to all physicians participating in post-emergency ward rounds. The summary was discussed at weekly ward-based meetings and displayed on ward notice boards. In addition, a project member gave junior doctors a summary sheet. 

Post-intervention (June-August 2021) data was collected on 375 patients over the three months. Compliance was calculated. The collected data was entered into Microsoft Excel and analyzed. Mean and standard deviation (SD) were calculated for quantitative variables and proportions for categorical variables. Since there was no revealing of the patient identity and no intervention to the patient (intervention was to the prescription practice), patient consent was not needed as per waiver by the ethics committee. Hospital authority consent was taken.

## Results

Table 1 shows the distribution of cases according to compliance in the patients who received antimicrobials. The compliance increased from 46% in the pre-intervention phase to 61% in the post-intervention phase. There were three cases in the pre-intervention phase in which antibiotic was indicated as per policy but not prescribed. There were four cases in the post-intervention phase in which antibiotic was indicated as per policy but not prescribed.

**Table 1:** Distribution of cases according to the number of patients on antimicrobials in compliance with policy

	Total number of study subjects	Total number of patients on antimicrobials	Number of patients who were on antimicrobials compliant with policy (%)	Number of patients who were on antimicrobials not compliant with policy (%)
Pre-intervention	375	225	104 (46)	121 (54)
Post-intervention	375	173	106 (61)	67 (39)

Table 2 shows the distribution of cases according to the indication of antimicrobials. In the pre-intervention phase in 268 patients, antimicrobial was not indicated out of which 118, i.e., 44% of patients received antimicrobials. However, in the post-intervention phase, in 265 patients, antimicrobial was not indicated out of which 65. i.e., 24.5% of patients received antimicrobials.

**Table 2:** Distribution of cases in whom antimicrobials were not indicated

	Number of patients in whom antimicrobials were not indicated	Number of patients on antimicrobial (%)	Number of patients not on antimicrobial (%)
Pre-intervention	268	118 (44)	150 (56)
Post-intervention	265	65 (24.5)	200 (75.5)

Table 3 shows the major clinical conditions and the prescription pattern of antimicrobials before and after the intervention.

**Table 3:** Clinical conditions with reference to antibiotic prescription URTI, upper respiratory tract infection; UTI, urinary tract infection; LRTI, lower respiratory tract infection; ASOM, acute suppurative otitis media

Sr. No.	Indications	Pre-intervention		Post-intervention	
		Number of patients	Patients given antibiotics (%)	Number of patients	Patients given antibiotics (%)
1	Dengue fever	71	48 (67.6)	60	17 (28.3)
2	Bacterial meningitis	04	04 (100)	02	02 (100)
3	Bacterial URTI	25	24 (96)	28	27 (96.4)
4	Acute febrile illness	15	10 (66.7)	15	07 (46.7)
5	UTI	18	18 (100)	14	14 (100)
6	Enteric fever	09	09 (100)	07	07 (100)
7	Dysentery	03	03 (100)	02	02 (100)
8	Bronchiolitis	07	06 (85.7)	09	03 (33.3)
9	Viral hepatitis	05	01 (20)	02	00 (00)
10	Rickettsia fever	01	01 (100)	03	03 (100)
11	Acute gastroenteritis	05	01 (20)	07	01 (14.3)
12	LRTI with bronchopneumonia	37	37 (100)	43	43 (100)
13	Viral fever	62	32 (51.6)	68	22 (32.4)
14	ASOM	02	02 (100)	03	03 (100)
15	Others	111	29 (26.1)	112	22 (19.6)

Dengue fever was the most common single infection noted in both phases where the unnecessary use of antibiotics declined from 68% in the pre-intervention phase to 28% in the post-intervention phase.

## Discussion

Infections due to antibiotic-resistant organisms are increasingly prevalent and represent a significant public health threat. Antibiotic overuse is a primary driver of this epidemic. Antibiotic stewardship is an essential means of limiting antibiotic resistance.

We observed that in the pre-intervention phase at our hospital, among 375 patients, 60% were on antimicrobials. In contrast, in the post-intervention phase, the proportion of patients on antibiotics reduced to 46.1%. Of those patients on antimicrobials, 46% complied with the policy in the pre-intervention phase, which increased to 61% in the post-intervention phase.

Of 375 patients in the pre-intervention and post-intervention phases, 268 and 265 did not require antibiotics prescription based on the policy guidelines. Among them, 44% and 24.5% received antibiotics in the pre-intervention and post-intervention phases. After the intervention program, the prescription of antibiotics without indication has reduced.

In a similar study by Thakkar et al. (2011), the intervention based on the plan-do-study-act approach to monitor and improve compliance with the antibiotic prescription policy improved the mean compliance from 30% to 71%. The study researchers from the UK hospital explained that their preliminary work that applied quality improvement methodology using monitoring, education, and feedback to support antimicrobial stewardship improved compliance with the policy [8]. Mol et al. study at the University Hospital Groningen, Netherlands, reported the results of a combined intervention strategy that involved updating antimicrobial prescription guidelines and disseminating them in paperback and electronic formats. The credibility of the antimicrobial prescription guidelines was also improved in consultation with the users. The study did not involve compulsory measures and observed that compliance increased from 67% to 86% [9]. The benefit of the intervention programs for judicious use of antibiotics has been reported by similar studies [10-14].

In the Indian context, the Chandy et al. study from South India suggested that the containment of highly irrational use of antibiotics was possible through active guideline dissemination. The authors recommended that the stakeholders and policymakers play an active role in developing antibiotic usage guidelines, ensuring the broader dissemination of the antibiotic stewardship policy, and enabling grassroots-level access through computer networks to contain non-judicious antibiotic use [15]. Recently, a study from New Delhi, India, reported that educational interventions regarding antibiotic prescribing and its frequent monitoring improved patterns toward rational antibiotic prescription among residents with no negative impact on patient outcomes [16]. A recent study regarding antimicrobial stewardship programs from seven Latin American countries used a standardized score tool based on Joint Commission International Accreditation Standards and the Colombian Institute of Technical Standards and Certification. Although the study saw drop out of a few institutions due to COVID-19, the study conclusions reflected that the standardized score tool was helpful in the evaluation of specific areas of antimicrobial stewardship programs and led to tailored interventions for the participating hospitals, consequently improving the development of better antimicrobial stewardship programs in the institutions that underwent pre-intervention and post-intervention analysis. Another important finding from the study was that the intervention strategies led to monetary savings on antimicrobial costs [17]. Weragama et al. did a systematic review of pediatric antimicrobial stewardship concerning respiratory infections encountered in the emergency setting and concluded that there is strong evidence that antimicrobial stewardship programs are very effective in reducing the prescription of antibiotics. The systematic review suggested that further studies are needed to assess whether the evidence translates to equivalent clinical outcomes [18].

Limitations

As a practical quality improvement program in an applied service setting, it was not possible to exert experimental control over extraneous factors that may have contributed to the observed effect. Due to the COVID-19 pandemic, the intervention and post-intervention phases were delayed and were done from March to August 2021 with due permission from the ethics committee. Also, the analysis of each intervention phase was not done separately.

## Conclusions

Compliance with antibiotic policy was seen in 46% of the pre-intervention and 61% of the post-intervention phase. Awareness, education, and feedback as an intervention helped increase compliance. Continuous cycles of awareness and feedback help achieve patient care and safety goals. There is a need for constant assessment and feedback regarding antibiotic prescription and compliance with guidelines to prevent the development of drug resistance among microbes.
